# Supplementary material for: Microbiome-mediated fructose depletion restricts murine gut colonization by vancomycin-resistant Enterococcus
Source: Nat Commun. 2022 Dec 13;13:7718. doi: 10.1038/s41467-022-35380-5 (PMC9748033; doi:10.1038/s41467-022-35380-5)
Supplement: Supplementary file 1 — Supplementary Information File [file 41467_2022_35380_MOESM1_ESM.pdf]

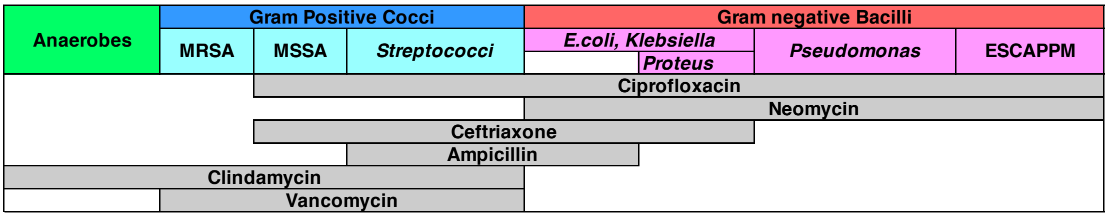

Supplementary Figure 1. Spectrum of the different antibiotics tested in Fig. 1

MRSA : Methicillin Resistant *Staphylococcus aureus*, MSSA : Methicillin Sensitive *Staphylococcus aureus*, ESCAPPM : bacteria with β-lactamase activity: *Enterobacter* spp., *Serratia* spp., *Citrobacter freundii*, *Aeromonas* spp., *Proteus* spp., *Providencia* spp., *Morganella morganii*. (Adapted from Wellingtonicu.com drug manual).

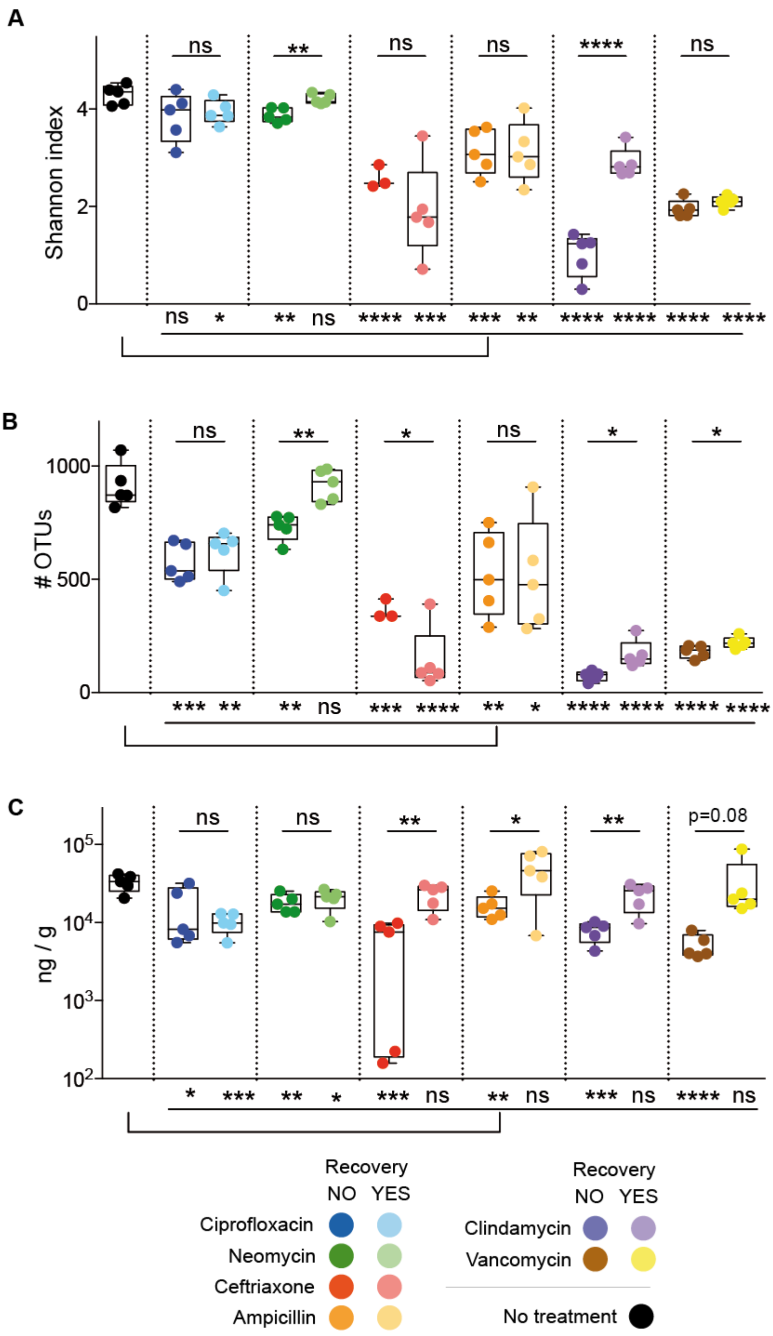

Supplementary Figure 2. Different antibiotics induces distinct changes in the microbiota diversity, richness and biomass. (A) Shannon index (diversity), ****p<0.0001,***p<0.001,**p<0.01,*p=0.0317; **(B)** number of Operational Taxonomical Units - OTUs (richness), ****p<0.0001,***p<0.001,**p<0.01,*p<0.05; **(C)** amount of DNA extracted (ng / g of faeces; Biomass),****p=8.2e-5,***p<0.001,**p<0.01,*p<0.05; detected in faecal samples from mice that received different antibiotic therapies as described in Fig. 1A. Two-sided t-test, ns – nonsignificant (p>0.05). N=5 mice per treatment, except for ceftriaxone without recovery (A-B) in which only 3 mice are shown because two of the mice contained too low amounts of DNA for sequencing. Statistical results refer to: below - the

comparison of each treated group of mice with the untreated group; above - the comparison of each group of mice treated with a specific antibiotic (before vs after the recovery period). Boxes extend from the 25th to 75th percentiles. The line within the boxes represents the median. Whiskers indicate the maximum and minimum values. Source data are provided as a Source Data file.

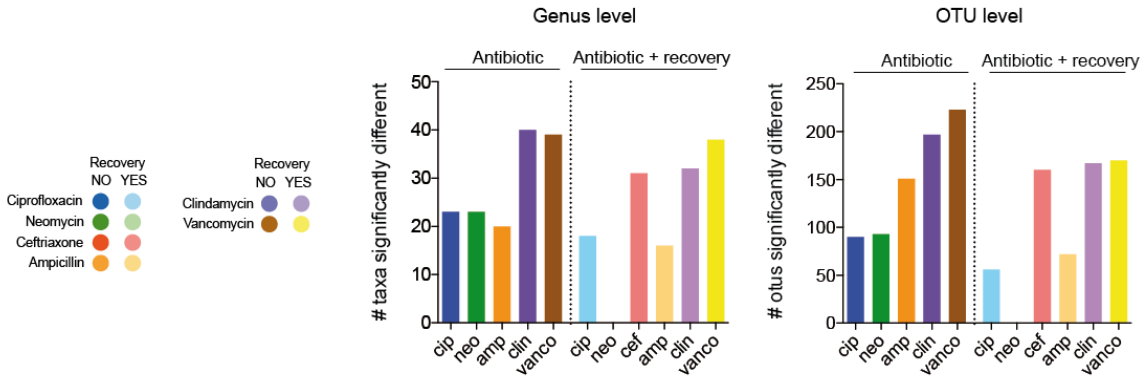

Supplementary Figure 3. Different antibiotics induce distinct changes in the microbiota at the genus and OTU level. Number of genera or Operational Taxonomical Units (OTUs) whose relative abundance significantly differs after antibiotic treatment administration as compared to untreated mice. Two-sided Wilcoxon rank-sum test & Benjamini-Hochberg ($q<0.05$). Groups of mice and antibiotics received are described in Fig. 1A. We could only analyse 3 samples of the 5 collected from ceftriaxone-treated mice (without recovery) since 2 of them contained very low amounts of DNA (Suppl. Fig. 2). This low number of mice led to nonsignificant results. For this reason, and to avoid misinterpretation when comparing the effect of different antibiotics on different taxa, we have not included the ceftriaxone group (without recovery) on the graph. The specific genera and specific OTUs (within the top most abundant) that were found to be significantly different among groups are shown in Suppl. Data Files 1 and 2. No treatment (NT), Ciprofloxacin (Cip), Neomycin (Neo), Ceftriaxone (Cef), Ampicillin (Amp), Clindamycin (Clin), Vancomycin (Vanco). Source data are provided as a Source Data file.

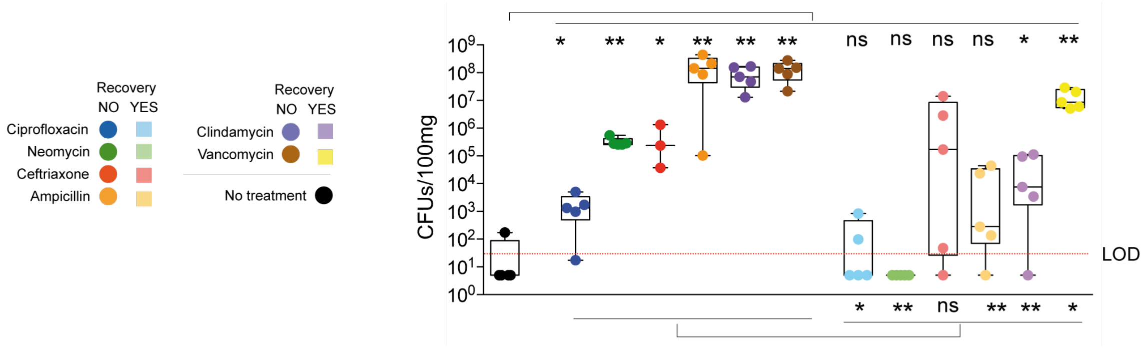

Supplementary Figure 4. Antibiotic treatments promote vancomycin-resistant *Enterococcus* (VRE) colonization of the caecum.

VRE caecal levels 2 days post-inoculation in the mice receiving the different treatments shown in Fig. 1. LOD = limit of detection. Points below the LOD indicate those mice in which we were not able to detect any VRE colony forming units (CFUs) after plating the caecal sample. *p<0.05, **p<0.008, ns – nonsignificant (p>0.086), two-sided Wilcoxon rank-sum test. N=5 mice per treatment, except in ceftriaxone without recovery in which N=3 mice. Statistical results refer to: above - the comparison of each treated group of mice with the untreated group; below - the comparison of each group of mice treated with a specific antibiotic (before vs after the recovery period). Boxes extend from the 25th to 75th percentiles. The line within the boxes represents the median. Whiskers indicate the maximum and minimum values. Source data are provided as a Source Data file.

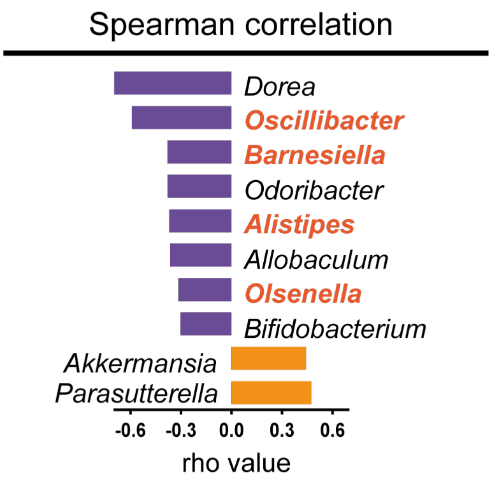

Supplementary Figure 5. Commensal bacterial genera significantly associated with VRE levels. Two-sided Spearman correlation test (q<0.05) between the faecal levels of commensal bacteria analyzed immediately before VRE inoculation and the faecal levels of VRE, 2 days

post-VRE inoculation. The bars represent the rho values. Purple colors represent negative correlations (high levels of the bacteria are associated with low levels of VRE) while orange colors represent positive correlations (high levels of the bacteria are associated with high levels of VRE). The red font denotes genera that could be isolated. Only abundant genera (median >0.4% in untreated mice, see methods) are included in the figure. Correlation analysis for all taxa are shown in Supp. Data File. 3. Source data are provided as a Source Data file.

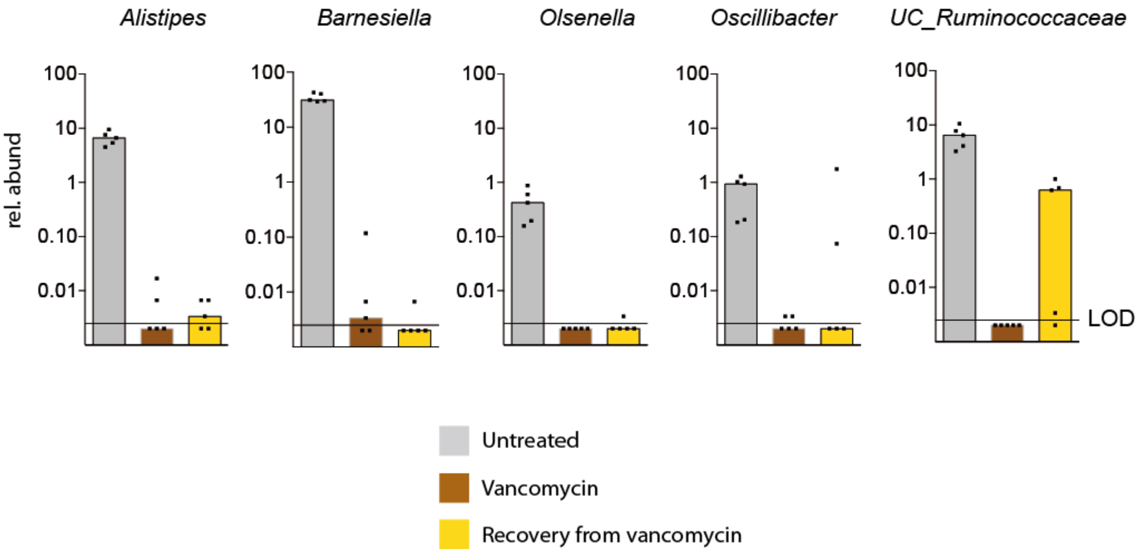

Supplementary Figure 6. Levels of the bacteria found to be associated with protection against VRE and that we were able to isolate. The relative abundance (rel.abund) at the genus level of each bacterium in the group of untreated mice, mice treated with vancomycin and mice allowed to recover from vancomycin treatment for 2 weeks is shown. Samples are derived from the experiment shown in Fig. 1. UC=unclassified. *UC_Ruminococcaceae* could only be classified at the family level using 16s rRNA sequencing. LOD: limit of detection. Samples in which these bacteria could not be detected are shown as points under the limit of detection. N=5 mice per group. Bars indicate the median. Source data are provided as a Source Data file.

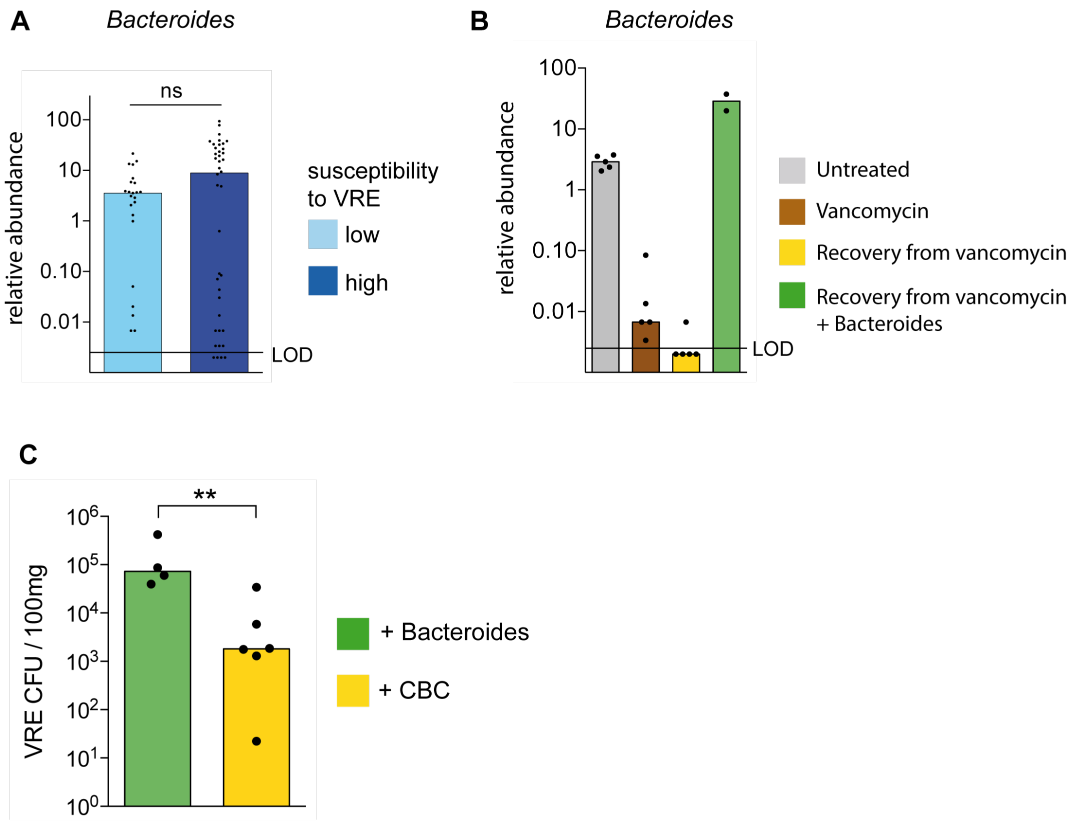

Supplementary Figure 7. *Bacteroides* is not associated with protection and does not restrict VRE intestinal colonization as compared to the bacterial consortium (CBC). (A) *Bacteroides* abundance in faecal samples from the mice described in Fig. 2A. **(B)** *Bacteroides* abundance of faecal samples described in Fig. 1 (vancomycin treatment) and in mice treated with vancomycin that received *Bacteroides* after stopping antibiotic treatment. **(C)** Mice were treated during one week with vancomycin. One day after antibiotic cessation, mice received during 3 consecutive days CBC or a *Bacteroides* isolate through oral gavage. Two weeks after stopping antibiotic treatment, a faecal sample was collected immediately before VRE inoculation to check in one representative mouse from each cage that *Bacteroides* was able to colonize the gut as shown in (B). CBC capability of colonizing the intestinal tract of vancomycin treated mice is shown in Suppl. Fig. 9. Two days post-VRE inoculation, VRE colony forming units (CFUs) were detected in faeces. **p=0.0095, ns – nonsignificant (p=0.318), two-sided Wilcoxon rank-sum test. N=23 and 40 mice per group in (A); 5,5,5, and 2 mice per group in (B), 4 and 6 mice per group in (C). Bars indicate the median. LOD: Limit of detection. Source data are provided as a Source Data file.

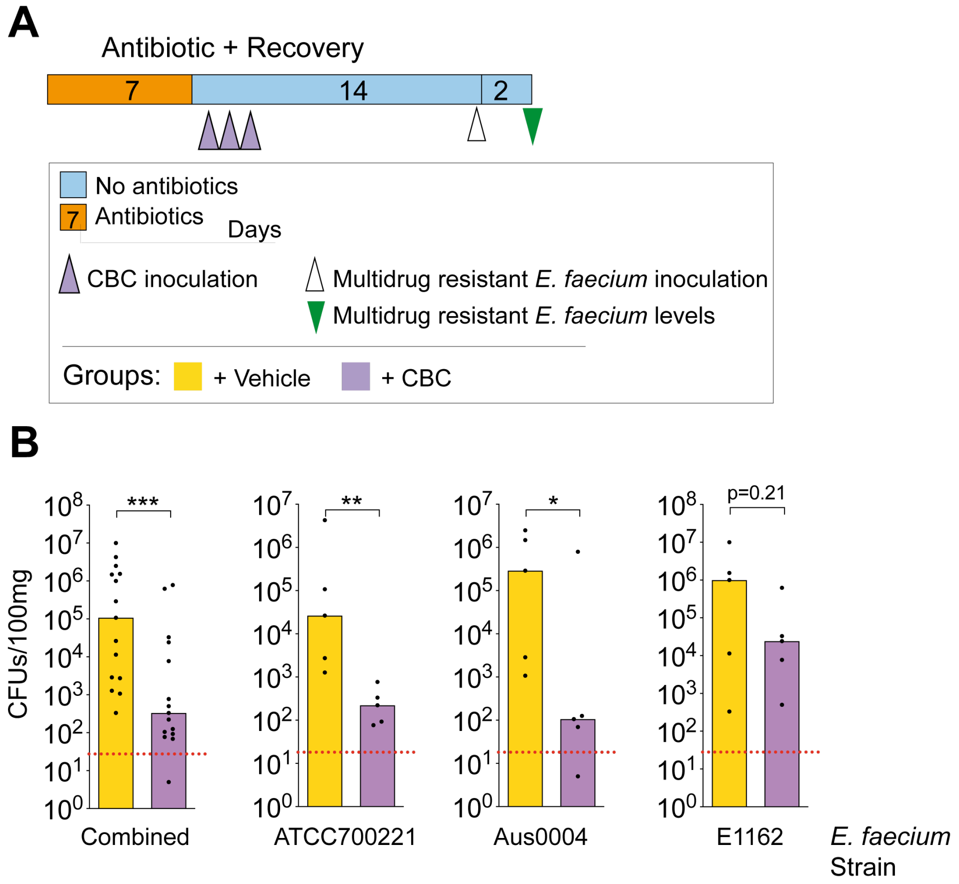

Supplementary Figure 8. The commensal bacterial consortium (CBC) restricts gut colonization of several multidrug-resistant *Enterococcus* strains. (A) Schematic representation of the mouse model used for testing the effect of CBC on colonization by several multidrug-resistant (MDR) *Enterococcus* strains. Mice were treated during one week with vancomycin. One day after antibiotic cessation, mice received during 3 consecutive days CBC through oral gavage. Two weeks after stopping antibiotic treatment, different strains of multidrug-resistant (MDR) *Enterococcus*, including two that are resistant to vancomycin (ATCC70021 and AUS0004), were inoculated through oral gavage. Two days post MDR-*E. faecium* inoculation (p.i.), colony forming units (CFUs) of the inoculated strains were detected in faeces. As control, a group of mice received the vehicle for bacteria administration (PBS-GC) instead of CBC. **(B)** Levels of MDR-*E. faecium* strains in faeces. N= 5 mice per group. Combined: results from the 3 strains were combined in one graph. The ATCC70021 strain was the strain used in the previous experiments. ***p=0.0007, **p<0.004, *p=0.028, One-sided Wilcoxon rank-sum test. Source data are provided as a Source Data file.

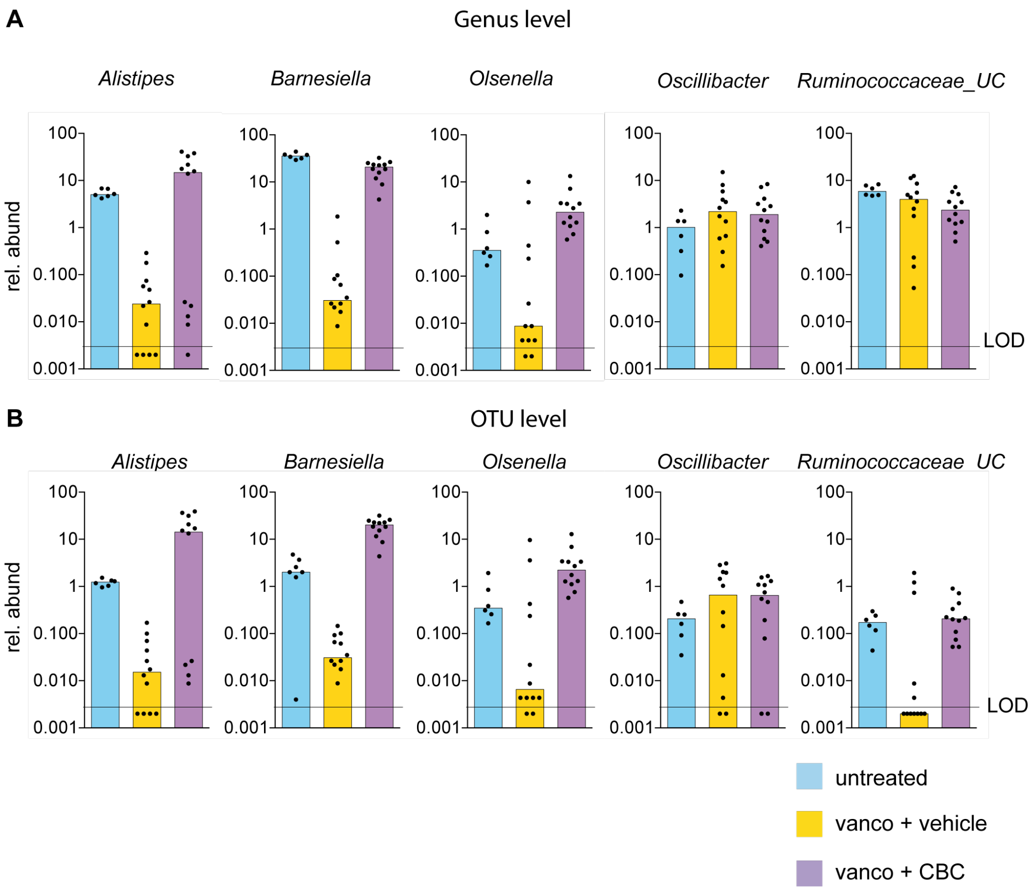

Supplementary Figure 9. Restoration of bacterial taxa lost after vancomycin treatment through the administration of the commensal bacterial consortium (CBC). Relative abundance (rel.abund) at the genus **(A)** or Operational Taxonomical Unit (OTU) **(B)** level of those taxa depleted after vancomcyin treatment and whose recovery was induced by the administration of CBC. The OTU level represents the OTUs to which the particular isolate administered belongs to. The groups of mice indicated in each graph are mice that did not receive any treatment, mice treated with vancomycin and allowed to recover 2 weeks after stopping the antibiotic treatment (vanco+vehicle) and vanco+CBC: same as vanco+vehicle but, in this case, mice received CBC instead of PBS-GC during 3 consecutive days, starting one day after stopping the antibiotic treatment. N=8, 12 and 12 mice per group. Bars represent the median. LOD: Limit of detection. UC: unclassified. Source data are provided as a Source Data file.

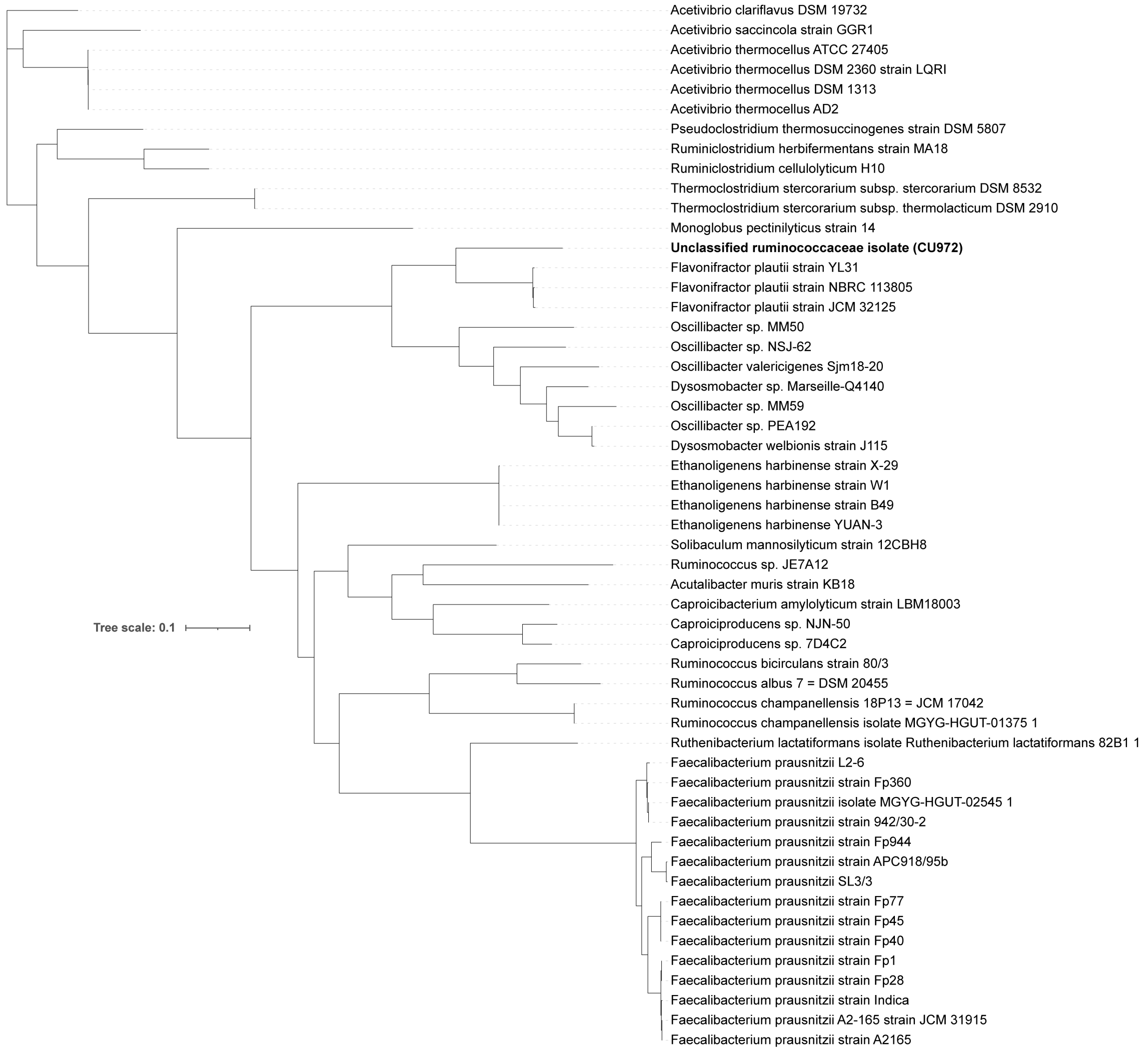

Supplementary Figure 10. The unclassified *Ruminococcaceae* isolate is included within the clade of the genus *Flavonifractor*. Phylogenetic tree was constructed with the core-genome of 52 reference genomes of the families *Ruminococcaceae*/*Oscillospiraceae* plus the isolate of interest (in bold font).

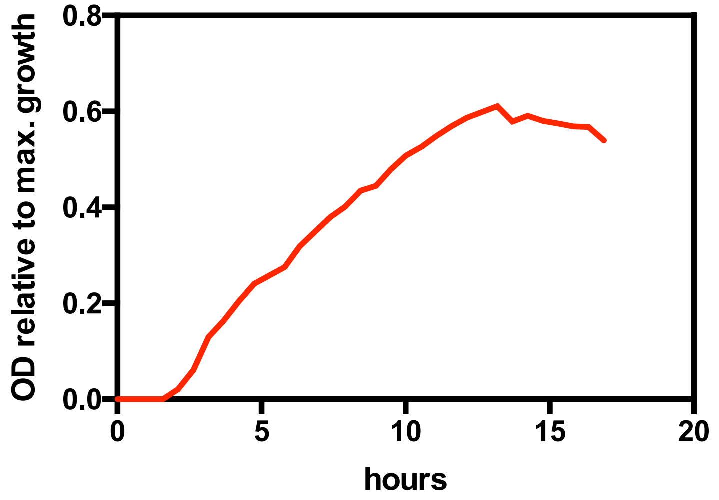

Supplementary Figure 11. ***Olsenella*** **is able to grow using fructose as carbon source.** Optical density values obtained in a pure culture of the *Olsenella* isolate grown under anaerobic conditions at 37 ºC. The culture was performed using a Biolog plate (see methods). The Optical density values are those obtained in the well containing fructose relative to the optical density obtained with the carbohydrate that promotes the highest growth of *Olsenella* (i.e. mannose). Source data are provided as a Source Data file.

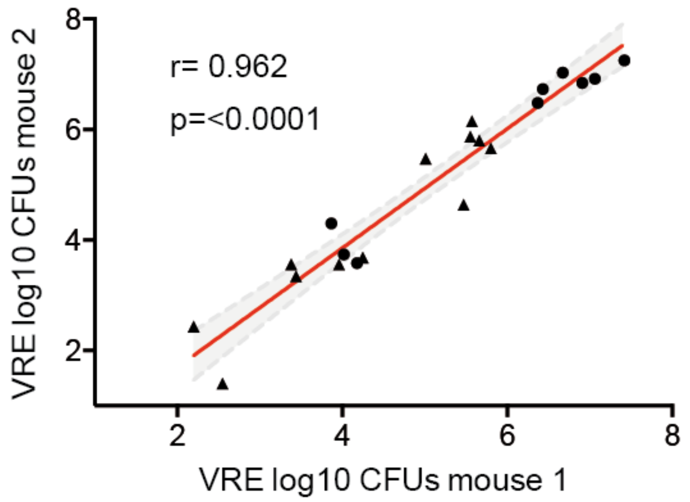

Supplementary Figure 12. Mouse co-housed in the same cage are equally susceptible to vancomycin-resistant ***Enterococcus*** **(VRE) intestinal colonization.** Mice were co-housed so that 2 mice were housed in the same cage. Co-housed mice were treated with vancomycin. A group of the co-housed mice received CBC during 3 consecutive days starting one day after stopping antibiotic treatment (triangles), while the other mice received PBS-GC instead (circles). Two weeks after stopping antibiotic treatment, mice were placed in new cages (one

mouse per cage) and received through oral gavage 10^6 VRE colony forming units (CFUs). Two days after, the levels of VRE were quantified in faeces. The graph shows the correlation between the levels of VRE of pair of mice that had been co-housed in the same cage. P and r values were obtained using the two-sided Pearson correlation analysis on the $\log_{10}$ VRE CFUs, p=1.2e-12. The line represents the linear regression mean and the grey shadow the 95% CI. N=21 pairs of mice. Source data are provided as a Source Data file.

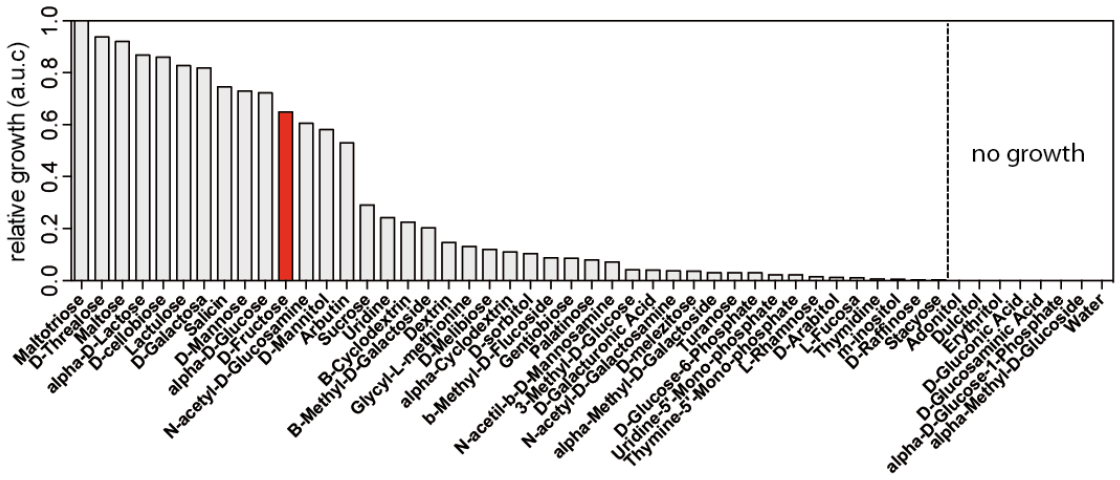

Supplementary Figure 13. Fructose is one of the carbohydrates that support vancomycin-resistant *Enterococcus* (VRE) growth to a higher extent *in vitro*. VRE was grown under anaerobic conditions at 37 ºC on a biolog plate (see methods). The optical density was measure during 16 h. The graph shows the area under the curve (a.u.c) of the growth curves obtained. The values are relative to the sugar that produced the maximum a.u.c (i.e. maltotriose). Within the carbon sources present in the biolog plate, only the sugars are shown. Source data are provided as a Source Data file.

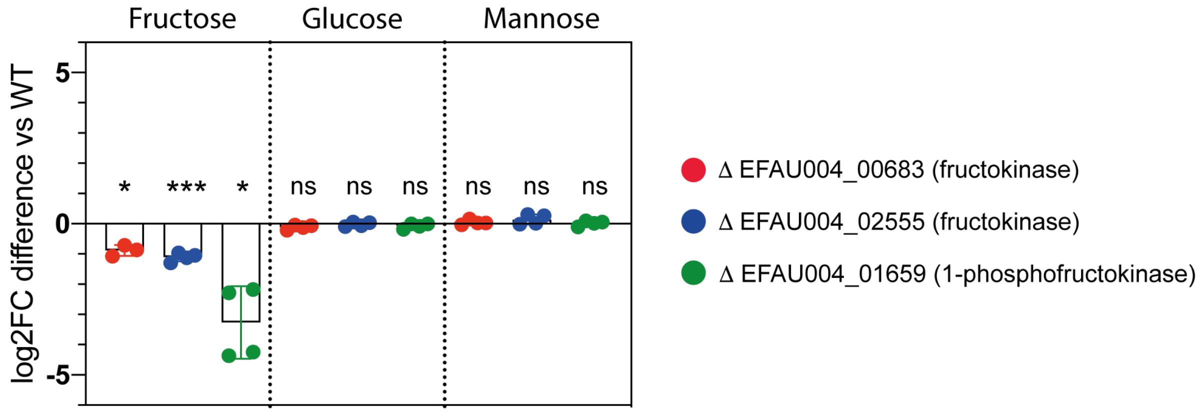

Supplementary Figure 14. Deletion of vancomycin-resistant *Enterococcus* (VRE)-encoded fructokinases impairs VRE growth using fructose as carbon source. VRE was grown in minimal media M1 supplemented with fructose, glucose or mannose. Cultures were grown for 24 hours and the optical density was monitored every 20 min. The area under the growth curve (AUC) was calculated for the mutants and wild-type (WT) strain. The figure shows the log2 fold change (FC) difference between the AUCs obtained with a mutant strain vs the WT. A negative value indicates lower growth of the mutant as compared to the WT. Two-sided one-sample t-test compared to 0 (the value representing no differences in growth between WT and mutant strains), ***p=0.0006, *p<0.014, ns- nonsignificant (p>0.058). Bargraphs represent the average, whiskers indicate the SEM. The data combines two independent experiments with two replicates in each experiment. Source data are provided as a Source Data file.

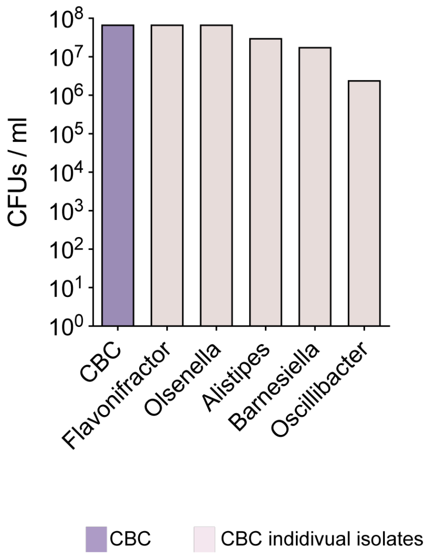

Supplementary Figure 15. Growth of commensal bacterial consortium strains *ex vivo*. CBC strains were grown individually or in combination at 37 ºC in anaerobic conditions in filtered supernatants of murine caecal contents from mice that were allowed to recover for two weeks after vancomycin treatment. After 24 h, dilutions were plated in columbia blood agar plates and colony forming units (CFUs) were quantified after 3 days, except *Oscillibacter* that was quantified after 6 days due to its slower growth. Source data are provided as a Source Data file.

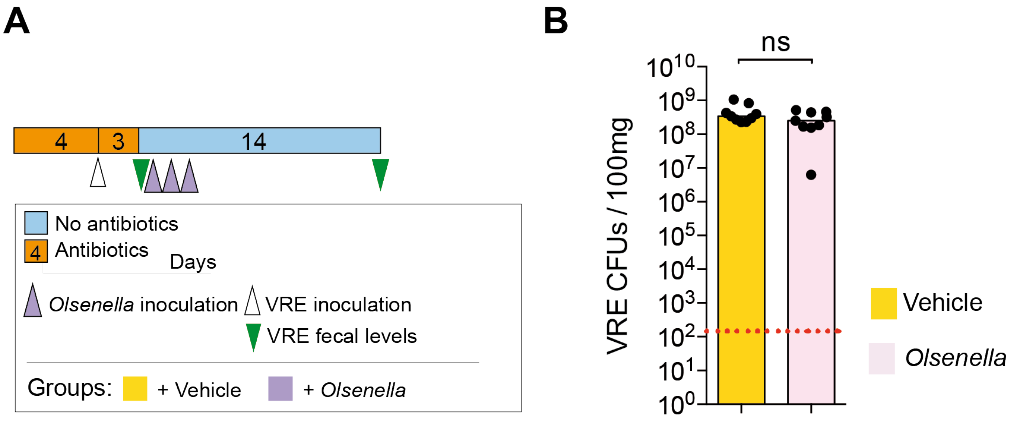

Supplementary Figure 16. VRE intestinal colonization before *Olsenella* inoculation. (A) Schematic representation of the mouse model used for testing the effect of *Olsenella* administration after VRE intestinal colonization. Mice were treated with vancomycin for 4 days. Subsequently, mice were inoculated with 10^6 VRE colony forming units (CFUs) by oral gavage. Three days after VRE inoculation the antibiotic treatment was stopped. One day after

antibiotic withdrawal, a faecal pellet was collected immediately before to the administration of *Olsenella* to half of the mice. **(B)** VRE levels of the faecal pellet collected immediately before *Olsenella* or Vehicle (PBS-GC) administration. ns – nonsignificant (p=0.258), two-sided Wilcoxon rank-sum test. N=9 mice per group. Bars represent the median. VRE faecal levels of the day 14 post-antibiotic cessation are shown in Fig. 5C. Source data are provided as a Source Data file.

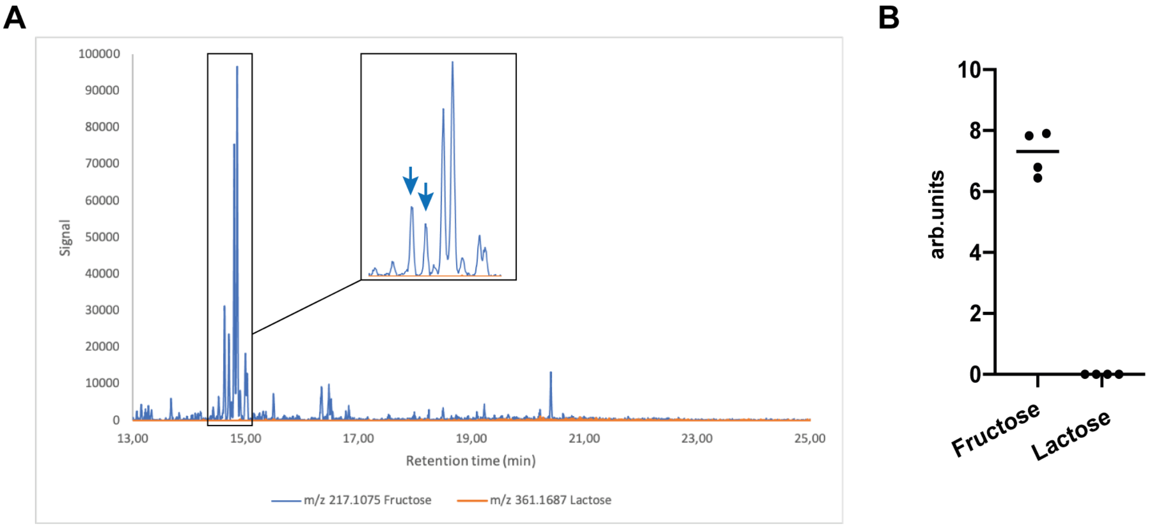

Supplementary Figure 17. Lactose could not be detected in caecal contents from mice that recovered from vancomycin treatment. Caecal samples were analyzed by GC-QToF to detect the presence of fructose and lactose. **(A)** The chromatogram obtained for one of 4 analyzed samples at the extracted m/Z of fructose (blue) and of lactose (orange). Two clear peaks (arrows), correponding to the two isomers obtained after the derivatization of fructose, with the retention time and mass of fructose, could be detected in the sample. However, no peak could be detected at the expected lactose mass at any retention time. **(B)** Levels of fructose or lactose in arbitrary units (arb. units) detected in the 4 analyzed samples.

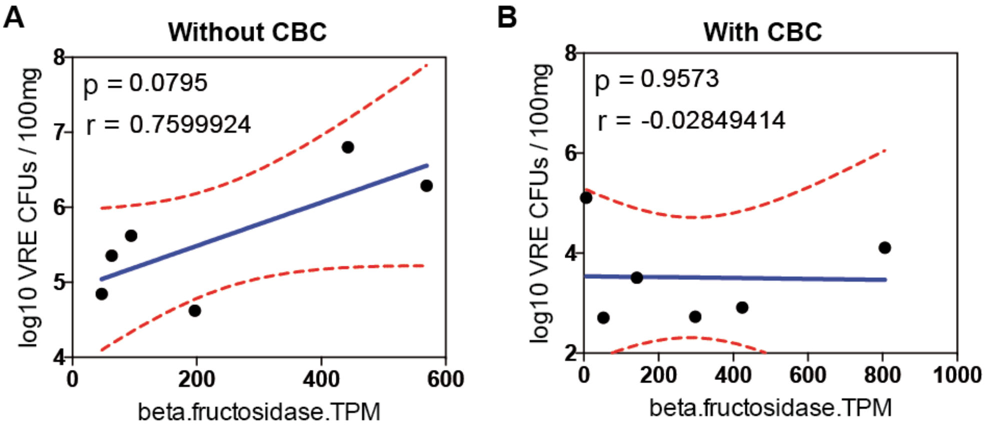

Supplementary Figure 18. Association between the levels of beta-fructosidase and vancomycin-resistant *Enterococcus* (VRE) levels. Correlation between the caecal levels of beta-fructosidases expressed by the microbiome and the VRE faecal levels in co-housed colonized mice 2 days after VRE inoculation (see methods). Expression of beta-fructosidases was analyzed 2 weeks after stopping the administration of vancomycin treatment in mice that received the bacterial consortium (CBC) (B) or received the bacterial vehicle instead (A). P and r values were calculated using the two-sided Pearson correlation test. VRE CFUs are in log10 scale so that both variables would follow a normal distribution. N=6 mice. The blue line represents the linear regression mean and the red lines represent the 95% CI. Source data are provided as a Source Data file.
