# Supplementary material for: Microbiome-mediated fructose depletion restricts murine gut colonization by vancomycin-resistant Enterococcus
Source: Nat Commun. 2022 Dec 13;13:7718. doi: 10.1038/s41467-022-35380-5 (PMC9748033; doi:10.1038/s41467-022-35380-5)
Supplement: Supplementary file 3 — Description of Additional Supplementary Files [file 41467_2022_35380_MOESM3_ESM.pdf]

Description of Additional Supplementary Files

SUPPLEMENTARY DATA FILES

Supplementary_Data_File_1.xlsx: Significant differences in the relative abundance of bacterial genera between untreated mice and mice that received antibiotics.

Supplementary_Data_File_2.xlsx: Taxonomic classification and statistical analysis of the OTUs shown in Fig. 1C.

Supplementary_Data_File_3.xlsx: Spearman correlation analysis between VRE faecal levels & relative abundance of bacterial genera identified in faeces.

Supplementary_Data_File_4.xlsx: Statistics of the sequenced genomes of CBC isolates.

Supplementary_Data_File_5.xlsx: Number of sequences analysed of each transcriptome from each mouse.

Supplementary_Data_File_6.xlsx: Primers and constructions generated to obtain the fructokinase VRE mutants.

Supplementary_Data_File_7.xlsx: Access codes for sequences generated in this study that have been deposited in public repositories.

Supplementary_Data_File_8.xlsx: Additional KOs (>1log_2FC) whose expression was increased upon CBC administration.

Supplementary_Data_File_9.xlsx: Tables containing the abundance of commensal bacteria identified through 16s rRNA sequencing in mice, the VRE colonization levels of antibiotic treated mice and bacterial gene expression levels of analyzed mice.
