# Supplementary material for: Microbiome-mediated fructose depletion restricts murine gut colonization by vancomycin-resistant Enterococcus
Source: Nat Commun. 2022 Dec 13;13:7718. doi: 10.1038/s41467-022-35380-5 (PMC9748033; doi:10.1038/s41467-022-35380-5)
Supplement: Supplementary file 13 — Reporting Summary [file 41467_2022_35380_MOESM13_ESM.pdf]

nature research

Corresponding author(s): Carles Ubeda

Last updated by author(s): 27/11/22

Reporting Summary

Nature Research wishes to improve the reproducibility of the work that we publish. This form provides structure for consistency and transparency in reporting. For further information on Nature Research policies, see our Editorial Policies and the Editorial Policy Checklist.

Statistics

For all statistical analyses, confirm that the following items are present in the figure legend, table legend, main text, or Methods section.

n/a	Confirmed	
☐	☒	The exact sample size (*n*) for each experimental group/condition, given as a discrete number and unit of measurement
☐	☒	A statement on whether measurements were taken from distinct samples or whether the same sample was measured repeatedly
☐	☒	The statistical test(s) used AND whether they are one- or two-sided<br>*Only common tests should be described solely by name; describe more complex techniques in the Methods section.*
☒	☐	A description of all covariates tested
☐	☒	A description of any assumptions or corrections, such as tests of normality and adjustment for multiple comparisons
☐	☒	A full description of the statistical parameters including central tendency (e.g. means) or other basic estimates (e.g. regression coefficient) AND variation (e.g. standard deviation) or associated estimates of uncertainty (e.g. confidence intervals)
☐	☒	For null hypothesis testing, the test statistic (e.g. *F*, *t*, *r*) with confidence intervals, effect sizes, degrees of freedom and *P* value noted<br>*Give P values as exact values whenever suitable.*
☒	☐	For Bayesian analysis, information on the choice of priors and Markov chain Monte Carlo settings
☒	☐	For hierarchical and complex designs, identification of the appropriate level for tests and full reporting of outcomes
☐	☒	Estimates of effect sizes (e.g. Cohen's *d*, Pearson's *r*), indicating how they were calculated

Our web collection on statistics for biologists contains articles on many of the points above.

Software and code

Policy information about availability of computer code

Data collection	Data from the Tecan Infinite spectrophotometer was acquired using the Magellan program v.6.6.
Data analysis	Quality of capillary electrophoresis obtained sequences was analyzed with Trev program from package Staden 2.0.<br>16s rRNA sequencing analysis: quality assessment of the obtained MiSeq illumina sequences was performed using printseq-lite v.0.20.4. Pair-end sequences were assembled using fastq-join v.1.1.2. Assembled sequences were processed using Mothur v1.39.1. Potentially chimeric sequences were removed using Uchime v.4.2.40. Sequences were grouped into OTUs using Vsearch v.2.9.0. Phylogenetic classification of sequences was performed with the Bayesian classifier algorithm implemented in Mothur v1.39.1.<br>Genome sequencing analysis: adaptor sequences were removed using Cutadapt v. 1.10. Sequences were then filtered by quality using UrQt v.1.0.18. Cleaned genomic data was assembled using SPAdes v. 3.7.1. Open reading frames (ORFs) were identified and annotated using PROKKA v.1.13. ORFs annotation was performed using HMMer v.3.1.2 using the KEGG database.<br>Metatranscriptomic analysis: Adaptor sequences and low-quality reads were removed using Cutadapt v. 1.10 and UrQt v.1.0.18. Ribosomal RNA sequences were removed mapping the sequences against the Short Ribosomal Subunit database from SILVA using bowtie v.2.2.9. Remaining reads were re-mapped against the Long Ribosomal Subunit database from SILVA and the Mouse reference genome from the NCBI reference repository also using bowtie v.2.2.9. Hits mapping any of both databases were discarded for further analysis. Transcriptomic sequences were assembled using SPAdes v. 3.7.1. ORFs were identified and annotated using MetaGeneMark v.1.0.1. ORFs were clustered at 90% identity using vsearch v.2.9.0 using the “cluster smallmem” option. Non-redundant (NR) ORFs were annotated using KEGG database and HMMer v.3.1.2. To calculate the average coverage and the total number of mapped reads per ORF, the filtered metatranscriptomic data was mapped against the formatted NR catalogue using bowtie2 v.2.2.9. In order to identify which transcripts were expressed by CBC isolates, cleaned metatranscriptomic data was mapped against the bacterial genomes using bowtie v.2.2.9. To identify fructanases, we used programs mentioned above and blast 2.10.0+.<br>Reclassification of the Unclassified Ruminococcaceae to Flavonifractor : The core-genome reconstruction of these genomes and the Unclassified_Ruminococcaceae of interest was done using the software OPSCAN v.0.1. The proteins encoded by the core-genome were individually aligned using the multi sequence alignment program MAFFT version 7.453 with the -linsi parameter. Non-informative regions of

the alignment were trimmed using trimAl v1.4.rev15 with the automated1 algorithm. The resulting alignments were then concatenated for each genome. The phylogenetic tree was inferred using IQ-TREE v.2.0.4. The phylogenetic tree was visualized with iTol v5.5.1
Additionaly for statistical analysis we used DeSeq2 algorithm v.1.24.0 and R version 3.3.2.
All the softwares used in this study are publicly available.

For manuscripts utilizing custom algorithms or software that are central to the research but not yet described in published literature, software must be made available to editors and reviewers. We strongly encourage code deposition in a community repository (e.g. GitHub). See the Nature Research guidelines for submitting code & software for further information.

Data

Policy information about availability of data

All manuscripts must include a data availability statement. This statement should provide the following information, where applicable:

- Accession codes, unique identifiers, or web links for publicly available datasets
- A list of figures that have associated raw data
- A description of any restrictions on data availability

The 16S rRNA sequencing data generated of antibiotic treated mice with and without recovery (corresponding to Fig. 1, Fig. 2A, Supp. Fig. 2, Supp. Fig.3, Suppl. Fig. 5, Suppl. Fig. 6, Suppl. Fig. 7) has been deposited in the European Nucleotide Archive (ENA) repository under accession code PRJEB40819 (https://www.ebi.ac.uk/ena/browser/view/PRJEB40819, Suppl. Data File 7). The 16S rRNA sequencing data generated of mice that received CBC or not (Fig. 2D-E, Supp Fig.9) has been deposited in ENA repository under accession code PRJEB40849 (https://www.ebi.ac.uk/ena/browser/view/PRJEB40849, Suppl. Data File 7). The genome sequences data generated has been deposited in the ENA repository under accession code PRJEB40866 (https://www.ebi.ac.uk/ena/browser/view/PRJEB40866, Suppl. Data File 7). The Transcriptomic sequences generated has been deposited in the ENA repository under accession code PRJEB40858 (https://www.ebi.ac.uk/ena/browser/view/PRJEB40858, Suppl. Data File 7). Tables containing the abundance of commensal bacteria identified through 16s rRNA sequencing in mice, the VRE colonization levels of antibiotic treated mice and bacterial gene expression levels of analyzed mice have been included in Suppl. Data File 9.
The following databases were used in this study: RefSeq genome database (https://www.ncbi.nlm.nih.gov/refseq/), SILVA database (https://www.arb-silva.de), PROKKA (https://github.com/tseemann/prokka), KEGG database (https://www.genome.jp/kegg/kegg2.html), Mouse reference genome v.38 from the NCBI reference repository (https://www.ncbi.nlm.nih.gov/genome/52), UNIPROT (https://www.uniprot.org).
Source data are provided with this paper.

Field-specific reporting

Please select the one below that is the best fit for your research. If you are not sure, read the appropriate sections before making your selection.

☒ Life sciences ☐ Behavioural & social sciences ☐ Ecological, evolutionary & environmental sciences

For a reference copy of the document with all sections, see nature.com/documents/nr-reporting-summary-flat.pdf

Life sciences study design

All studies must disclose on these points even when the disclosure is negative.

Sample size	Sample-size calculation was not performed since it was not possible to predict the magnitude of the variation between animals for a particular parameter based on our current knowledge. However, considering previous studies on colonization resistance using SPF mice (PMID31959968, PMID36153315), at least 5 mice per group were included in all in vivo experiments with SPF mice. This allowed us to detect statistically significant differences among groups of mice regarding the major factors evaluated (pathogen gut levels, differences in microbial taxa, transcripts and metabolites). For in vitro and ex vivo assays, sample size calculation was not performed. However, since we detected a lower variability between samples from the same group as compare to the in vivo studies and a lower number of samples is required to obtain statistically significant results when intragroup variability decreases, in these particular cases the number of samples that were included per group was at least 3. This number of samples was also used in a previous study (PMID36153315) identifying mechanism of colonization resistance against multidrug resistant pathogens when this low intragroup variability was detected. Thus number of samples allowed us, as in the previous mentioned study, to reach statistically significant results when differences in the variable under study were detected among groups of samples.
Data exclusions	In the results shown in Fig. 1, only 3 mice are included in the group that received ceftriaxone (without recovery) because in 2 of the mice the amount of extracted DNA was too low and no 16s rRNA amplification could be achieved. In the results shown in Fig. 4A, one mouse from the group that did not received the bacterial consortium was excluded from the analysis because after microbiome analysis we detected that this mouse had spontaneously recovered very high levels of Olsenella (>10%), the key bacteria in the CBC consortium. As expected, the co-housed mouse was highly resistant to VRE colonization (8x102 VRE CFUs / 100 mg). Including this mouse in the analysis could interfere with the results considering that the hypothesis to test was that restoration of CBC (specifically Olsenella) decreases the levels of fructose which confers resistance to VRE colonization.
Replication	Hypothesis generating experiments were performed once: metagenomic analysis to identify commensal bacteria associated with protection, metatranscriptomic and metabolomic analysis to identify bacterial functions and metabolites (i.e. fructose) associated with protection. Hypothesis testing experiments were performed twice: in vivo CBC and Olsenella administration to confirm their inhibitory effect against VRE, in vivo fructose administration to demonstrate the effect of fructose on VRE growth in vivo, ex vivo experiments to demonstrate the inhibitory effect of Olsenella and CBC and VRE growth restoration after an excess of fructose addition. When experiments were repeated, they yielded comparable results.
Randomization	All mice were randomized to avoid cage effects.
Blinding	Blinding was applied to the quantification of the CFUs in order to determine the levels of VRE in the different groups of samples.

Also blinding was applied to the RNA and DNA extraction and sequencing and the metabolomic sample preparation. However, for sequencing analysis blinding was not applied since the investigator needs to know to which group belongs each sample in order to perform the analysis.

Reporting for specific materials, systems and methods

We require information from authors about some types of materials, experimental systems and methods used in many studies. Here, indicate whether each material, system or method listed is relevant to your study. If you are not sure if a list item applies to your research, read the appropriate section before selecting a response.

Materials & experimental systems

n/a	Involved in the study
☒	☐ Antibodies
☒	☐ Eukaryotic cell lines
☒	☐ Palaeontology and archaeology
☐	☒ Animals and other organisms
☒	☐ Human research participants
☒	☐ Clinical data
☒	☐ Dual use research of concern

Methods

n/a	Involved in the study
☒	☐ ChIP-seq
☒	☐ Flow cytometry
☒	☐ MRI-based neuroimaging

Animals and other organisms

Policy information about studies involving animals; ARRIVE guidelines recommended for reporting animal research

Laboratory animals	Mice were maintained accordingly to the National guidelines (RD 53/2013), under protocols approved by University of Valencia Animal Care Committee describing experiments specific for this study. Experiments were done with 7-week-old C57BL/6J female mice directly purchased from Charles River laboratories (Figures 1, 2, 3, 4A, 4B and Supplementary Figures 2-7, 9, 12 and 18) or obtained from the offspring of C57BL/6J mice purchased from Charles River laboratories that were bred in our animal facility (Figures 4D, 4F, 5 and Supplementary Figures 8 and 16). Mice were housed with autoclave-sterilized food (a 1:1 mixture of 2014S Teklad Global diet and 2019S Teklad Global Extrused 19% Protein Rodent Diet from Envigo) and autoclave-sterilized water, except in the experiment shown in Figure 4D in which mice received also the Teklad diet TD05075.
Wild animals	The study did not involve wild animals.
Field-collected samples	The study did not involve samples collected from the field
Ethics oversight	All mouse procedures were performed in accordance with institutional protocol guidelines at the “Servei Central de Suport a la Investigació Experimental” at the University of Valencia. Mice were maintained accordingly to the National guidelines (RD 53/2013), under protocols approved by University of Valencia Animal Care Committee describing experiments specific for this study.

Note that full information on the approval of the study protocol must also be provided in the manuscript.
